# High-precision stereotactic irradiation for focal drug-resistant epilepsy versus standard treatment: a randomized waitlist-controlled trial (the PRECISION trial)

**DOI:** 10.1186/s13063-024-08168-9

**Published:** 2024-05-21

**Authors:** C. M. L. Zegers, A. Swinnen, C. Roumen, A. L. Hoffmann, E. G. C. Troost, C. J. J. van Asch, L. Brandts, I. Compter, E. M. T. Dieleman, J. B. Dijkstra, M. Granzier, M. Hendriks, P. Hofman, R. M. A. Houben, B. Ramaekers, H. E. Ronner, R. P. W. Rouhl, S. van der Salm, R. G. C. Santegoeds, J. J. Verhoeff, G. L. Wagner, J. Zwemmer, OEMG Schijns, A. J. Colon, D. B. P. Eekers

**Affiliations:** 1 Department of Radiation Oncology (Maastro), GROW Research Institute for Oncology and Reproduction, Maastricht University Medical Centre+, Maastricht, the Netherlands; 2 Department of Health Services Research, Care and Public Health Research Institute (CAPHRI), Faculty of Health Medicine and Life Sciences, Maastricht University, Maastricht, the Netherlands; 3 OncoRay - National Center for Radiation Research in Oncology, Faculty of Medicine and University Hospital Carl Gustav Carus, Technische Universität Dresden, Helmholtz-Zentrum Dresden-Rossendorf, Dresden, Germany; 4 Helmholtz-Zentrum Dresden-Rossendorf, Institute of Radiooncology-OncoRay, Dresden, Germany; 5 Department of Radiotherapy and Radiation Oncology, Faculty of Medicine and University Hospital Carl Gustav Carus, Technische Universität Dresden, Dresden, Germany; 6 German Cancer Consortium (DKTK), Partner Site Dresden, and German Cancer Research Center (DKFZ), Heidelberg, Germany; 7 National Center for Tumor Diseases (NCT), Partner Site, Dresden, Germany; 8 German Cancer Research Center (DKFZ), Heidelberg, Germany; 9 Faculty of Medicine and University Hospital Carl Gustav Carus, Technische Universität Dresden, Dresden, Germany; 10 Helmholtz Association/Helmholtz-Zentrum Dresden-Rossendorf (HZDR), Dresden, Germany; 11 Stichting Epilepsie Instellingen Nederland (SEIN), Zwolle, the Netherlands; 12 Department of Clinical Epidemiology and Medical Technology Assessment, Maastricht University Medical Center+, Maastricht, the Netherlands; 13 Department of Radiotherapy, Amsterdam UMC (AMC), Amsterdam, the Netherlands; 14 Department of Medical Psychology, Maastricht University Medical Center+, MHeNs School for Mental Health and Neuroscience, Maastricht, the Netherlands; 15 Academic Center for Epileptology Kempenhaeghe, Maastricht University Medical Center, Maastricht, the Netherlands; 16 Donders Institute for Brain, Cognition and Behaviour, Radboud University, Nijmegen, the Netherlands; 17 Department of Radiology and Nuclear Medicine, Maastricht University Medical Center, Maastricht, the Netherlands; 18 Care and Public Health Research Institute (CAPHRI), Maastricht University, Maastricht, the Netherlands; 19 Department of Clinical Neurophysiology, Amsterdam UMC, Amsterdam, the Netherlands; 20 School for Mental Health and Neuroscience, Maastricht University, Maastricht, the Netherlands; 21 Department of Neurology, Maastricht University Medical Center+, Maastricht, the Netherlands; 22 University Medical Center Utrecht (UMCU), Utrecht, the Netherlands; 23 Department of Radiation Oncology, UMC Utrecht, 3584 CX Utrecht, the Netherlands; 24 Department of Clinical Neurophysiology, Stichting Epilepsie Instellingen Nederland (SEIN), Heemstede, the Netherlands; 25 Department of Neurosurgery, Maastricht University Medical Center, Maastricht, the Netherlands; 26 Department of Epileptology, CHU Martinique, Fort-de-France, France

**Keywords:** Epilepsy, Radiosurgery, Stereotactic radiation therapy (SRT), MRI, Cognition

## Abstract

**Introduction:**

The standard treatment for patients with focal drug-resistant epilepsy (DRE) who are not eligible for open brain surgery is the continuation of anti-seizure medication (ASM) and neuromodulation. This treatment does not cure epilepsy but only decreases severity. The PRECISION trial offers a non-invasive, possibly curative intervention for these patients, which consist of a single stereotactic radiotherapy (SRT) treatment. Previous studies have shown promising results of SRT in this patient population. Nevertheless, this intervention is not yet available and reimbursed in the Netherlands. We hypothesize that: SRT is a superior treatment option compared to palliative standard of care, for patients with focal DRE, not eligible for open surgery, resulting in a higher reduction of seizure frequency (with 50% of the patients reaching a 75% seizure frequency reduction at 2 years follow-up).

**Methods:**

In this waitlist-controlled phase 3 clinical trial, participants are randomly assigned in a 1:1 ratio to either receive SRT as the intervention, while the standard treatments consist of ASM continuation and neuromodulation. After 2-year follow-up, patients randomized for the standard treatment (waitlist-control group) are offered SRT. Patients aged ≥ 18 years with focal DRE and a pretreatment defined epileptogenic zone (EZ) not eligible for open surgery will be included. The intervention is a LINAC-based single fraction (24 Gy) SRT treatment. The target volume is defined as the epileptogenic zone (EZ) on all (non) invasive examinations. The seizure frequency will be monitored on a daily basis using an electronic diary and an automatic seizure detection system during the night. Potential side effects are evaluated using advanced MRI, cognitive evaluation, Common Toxicity Criteria, and patient-reported outcome questionnaires. In addition, the cost-effectiveness of the SRT treatment will be evaluated.

**Discussion:**

This is the first randomized trial comparing SRT with standard of care in patients with DRE, non-eligible for open surgery. The primary objective is to determine whether SRT significantly reduces the seizure frequency 2 years after treatment. The results of this trial can influence the current clinical practice and medical cost reimbursement in the Netherlands for patients with focal DRE who are not eligible for open surgery, providing a non-invasive curative treatment option.

**Trial registration:**

Clinicaltrials.gov Identifier: NCT05182437. Registered on September 27, 2021.

**Supplementary Information:**

The online version contains supplementary material available at 10.1186/s13063-024-08168-9.

## Background

Epilepsy has an incidence of around ~ 200,000 patients in the Netherlands and is a great burden to many patients due to the unpredictability of the seizures, a low quality of life, and the increased mortality rate in patients with epilepsy [1–4]. The population of interest for this trial is the group of patients with chronic and focal (= localization related) drug-resistant epilepsy (DRE) who are not eligible for, or do not want to undergo, open resective surgery. In the Netherlands, patients are selected for epilepsy surgery after discussion in a multidisciplinary team in three regional epilepsy surgery working groups connected to three university hospitals (UMC Utrecht, Amsterdam UMC, Maastricht UMC +) resulting in a total of approximately 200–250 surgeries per year [5]. The postoperative seizure freedom rates (= curation) vary between 60 and 90% with epileptogenic lesions on MRI and lesion type as major predictive factors [6]. It is estimated that about 140–150 patients/year within the Netherlands with a suspected focal origin of seizures cannot be offered open surgery and will receive the indication for palliative treatment (deep brain or vagus nerve stimulation (DBS or VNS) and chronic anti-seizure medication (ASM). Seizures and chronic use of ASM cause different somatic and mental side effects and bring also psychosocial consequences, e.g., feelings of dependence, anxiety, depression, and stigma, each impacting patients’ quality of life. Furthermore, the associated yearly healthcare costs for society are expected to be more than the 2004 estimate of €9500 per patient [7–9].

In the PRECISION trial, adult patients with focal DRE, not eligible for surgery, are offered non-invasive linear accelerator (LINAC)-based stereotactic radiotherapy (SRT) with curative intent. SRT has been used to treat several types of neoplasms in the brain for several decades. LINAC-based SRT refers to delivering the dose in one or several treatment sessions. SRT has been proven safe and effective for high-precision radiation treatment of small target volumes, e.g., brain metastases, benign tumors such as meningioma, pituitary adenoma, and vestibular schwannoma, in particular for those locations not easily accessible for surgery [10, 11].

**Fig. 1:**
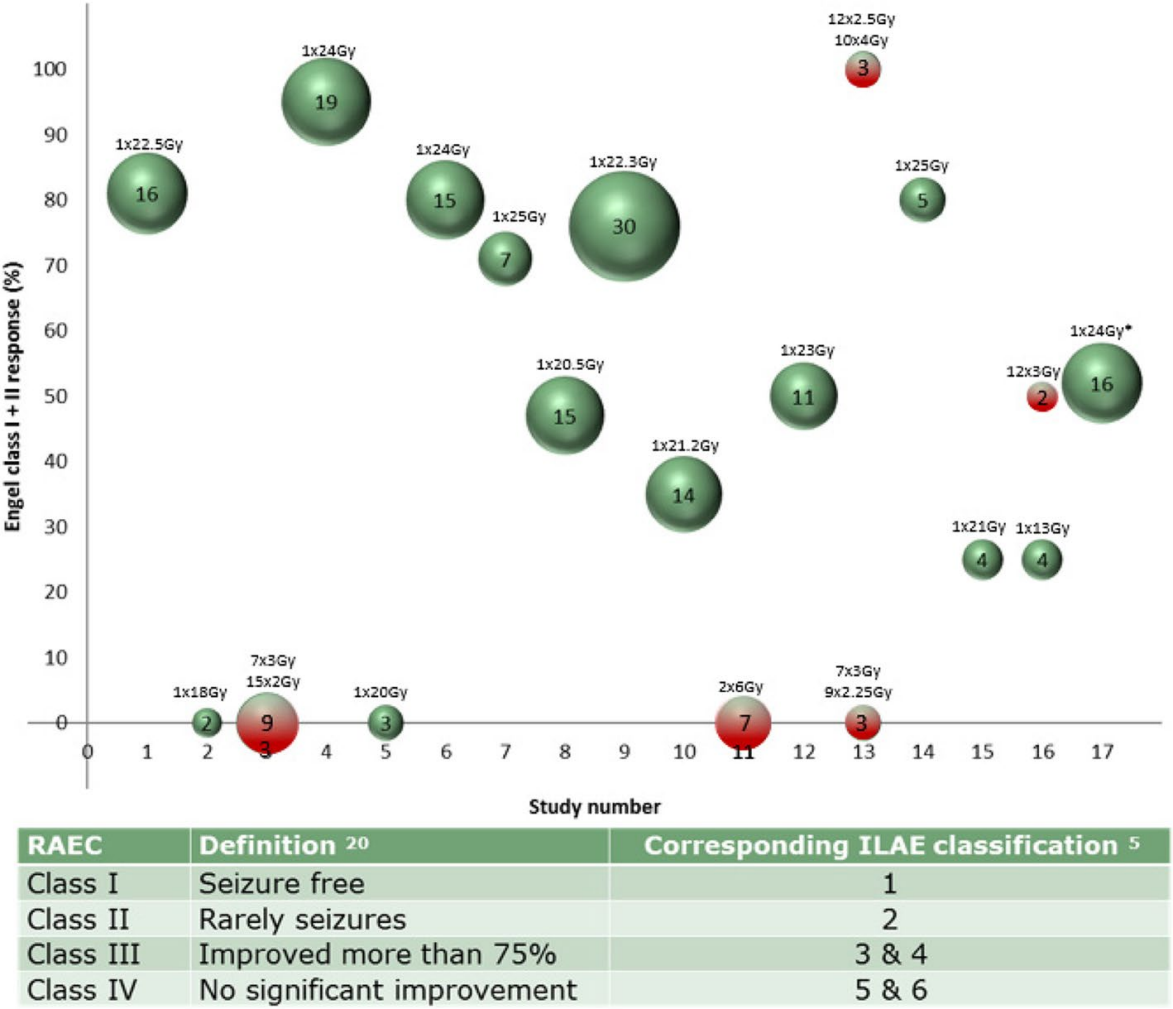
Adapted from Eekers et al. [17]. Summarizing the percentage of patients with a radiotherapy-adapted Engel class (RAEC) I or II. On the horizontal axis, the study numbers are given; on the vertical axis, the post-treatment RAEC outcome percentage of patients is plotted. The numbers given and the size of the respective bubble indicate the number of patients included in each study. The green color indicates a single fraction, while red highlights multiple fractions. The prescribed dose (number of fractions times the mean fraction dose) is given above each bubble. * = the ROSE trial [6]

Several publications (level 2 evidence) have shown the potential value of SRT in patients with DRE [12–16]; however, no level 1 evidence was provided enabling guideline development. The systematic review of Eekers et al. has shown that SRT resulted in a significant seizure cure or reduction in 58% of the 170 included patients, within 2 years after treatment [17]. However, all the evidence from the studies described in the review are from cohort studies with a relative low number of patients, who had different pathological lesions as a cause for their epilepsy. In addition, the radiotherapy treatment schedules were diverse (Fig. 1). Interestingly, the ROSE trial, randomizing between open surgery and SRT, has demonstrated a seizure remission of 52% in the radiotherapy group after 2 years with the proportion of seizure-free patients still increasing with a longer follow-up up to 74% after 3 years [4]. At the moment, there is neither a randomized study available comparing seizure outcome after SRT with the current standard of care treatment for this population nor a cost-effectiveness analysis. Therefore, the current trial is necessary to show the relative (cost-)effectiveness of SRT in comparison to the standard care for patient’s ineligible for surgery.

The primary objective of this phase 3 randomized waitlist-controlled trial is to determine whether stereotactic radiotherapy (SRT) reduces the seizure frequency resulting in a reduction of at least 75% (radiotherapy-adapted Engel classification (RAEC) I–III [17]) 2 years after treatment in patients with focal DRE and not eligible for open surgery, when compared to standard of care. Secondary objectives are to assess quality of life (QoL) and neuro-cognition after SRT, (serious) adverse effects, and ASM use and to investigate the cost-effectiveness of SRT compared to standard of care.

## Methods/design

### Study design

**Fig. 2:**
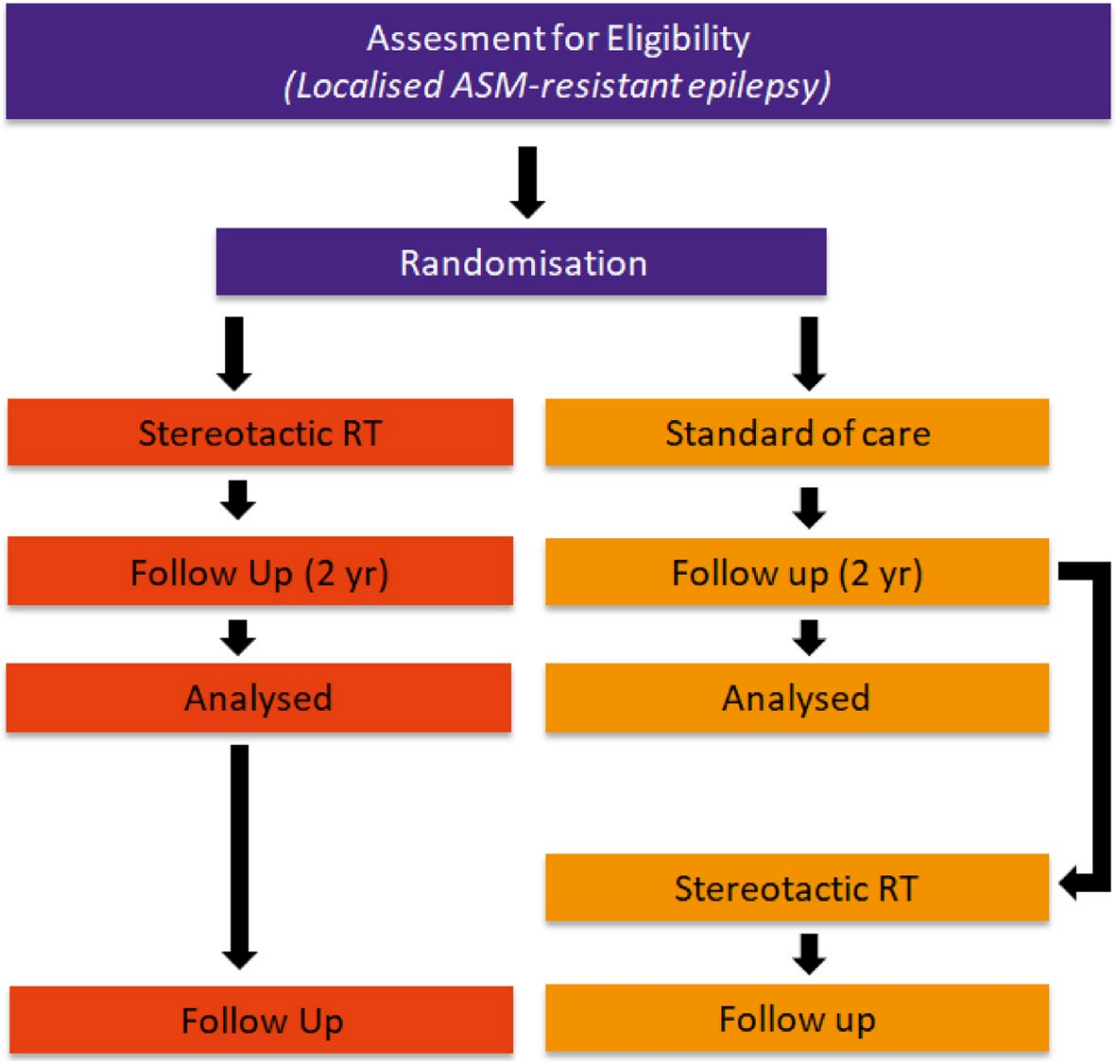
Schematic overview of the study design (RT, radiotherapy; ASM, anti-seizure medication)

Patients will be included for the superiority randomized waitlist-controlled PRECISION trial in the department of radiation oncology (Maastro), Maastricht, the Netherlands. Patients will be randomized in a 1:1 ratio between SRT and current standard care (waitlist-control group), where the latter includes ASM and neuromodulation (i.e., DBS or VNS). The waitlist-controlled group will be offered SRT after the follow-up period of 2 years (optional), if the patients still meet the inclusion and exclusion criteria (Fig. 2). We hypothesize that SRT is a (cost)effective method to alter the epileptogenic cerebral tissue to yield a reduction in seizures and possibly cure after 2 years, with a significant increase in the patients’ quality of life [7, 10].

### Sample size calculation

Participants will be randomized between SRT and standard of care at a ratio of 1:1. We expect that 50% of the patients in the SRT group will reach RAEC I–III after 2-year follow-up compared to at best 15% in the standard of care group [17, 18]. Sample size calculation is based on a two-sided *Z*-test for proportions with pooled variance and continuity correction. Significance level alpha is set at 0.05 and power at 0.90. Dropout is set at 10%. A total number of 84 patients is needed for the analysis, randomized equally to the SRT group and the standard of care group. After correction for drop-out, the total number of patients becomes 94, with 47 patients randomized to each treatment arm.

### Recruitment

In the Netherlands, there are 3 multidisciplinary comprehensive epilepsy-surgery conference groups, in 3 different regions. The neurologists of the 3 conference groups were informed by the researchers about the PRECISION trial, and each group has a contact person for PRECISION. The neurologists can find more details of the PRECISION trial (e.g., in- exclusion criteria) on the website of Maastro. Potential patients from all conference groups will be evaluated by the multidisciplinary comprehensive epilepsy surgery conference of Maastricht UMC + and Kempenhaeghe (AWEC), consisting of inter alia, a neurologist, neurosurgeon, radiologist, neuropsychologist, and a radiation oncologist, for eligibility. In case the patient meets the inclusion criteria, the treating physician will ask the patients' permission to be contacted by Maastro. Informed consent and recruitment will be performed by the radiation-oncologists from Maastro. The recruitment period of the trial is 3.5 years, with an estimated accrual rate of 2–3 patients per month.

### Inclusion and exclusion criteria

Patients with focal DRE not eligible for surgery are eligible for this study. In addition, all patients must meet all the following criteria:

1. Age ≥ 18 years
2. Written informed consent is given according to International Conference on Harmonization Good Clinical Practice (ICH-GCP) and national/local regulations
3. The patient is willing to use contraception during the SRT treatment and for at least 30 days following the SRT therapy
4. The patient or caretaker is able to keep an epilepsy diary
5. The patient has a diagnosis of epilepsy established by a dedicated neurologist
6. The patient had at least 3 focal-onset seizures over a 3-month period despite two or more antiseizure medication trials (according ILAE Task Force on therapeutic strategies)
7. Video electroencephalography and work-up in the multidisciplinary team to determine a well-circumscribed seizure focus is available
8. There is evidence (e.g., 3 T-MRI or a clear SEEG delineation) of the anatomic region to be targeted with SRT, correlating with the seizure focus;
9. A functional MRI to lateralize language or localize visual, motor, and/or sensory eloquent cortex has been performed in selected patients (if the lesion is expected to be located, based on anatomy, in eloquent areas)
10. The patient has completed a standard battery of neuropsychological testing
11. The patient been deemed an appropriate candidate for stereotactic radiosurgery by a dedicated radiotherapist, neurosurgeon, and epileptologist and referred for the study by one of the Dutch regional multidisciplinary epilepsy surgery working groups
12. Patients that were rejected for surgery in an earlier stage can participate in the trial if the last change of the VNS/DBS settings were more than 1 year ago or VNS/DBS was not (yet) tried.

The most important exclusion criteria are as follows: pregnancy, prior cranial radiotherapy, or a clinically significant and uncontrolled major other medical condition(s). In addition, if a radiation treatment plan without exceeding the constraints for the organs at risk is not feasible, patients will be excluded from this study.

### Study treatment

Patients in the intervention arm will receive LINAC-based stereotactic radiotherapy (SRT). The target volume is defined as the epileptogenic zone (EZ) on all clinically available (non) invasive examinations in combination with the dedicated MRI and planning CT images acquired within the trial. A single fraction SRT is given with a prescribed isotoxic dose of 24 Gy to the 100% surrounding isodose. Dose is depending on the proximity and maximum tolerable dose to the radiosensitive organs at risk, the EZ volume, and location (e.g., eloquent areas) resulting in a V12Gy ≤ 10.9 cc reducing the risk on radiation necrosis [19]. Therefore, the standard prescription dose will be 24 Gy but can be lowered to 18 Gy based on the V12Gy.

The waitlist-control group (= comparator) will be composed as follows. In case a patient is rejected for open epilepsy surgery and randomized to the waitlist, he/she will receive a proposal for palliative treatment options, i.a. vagal nerve stimulation (VNS) or deep brain stimulation (DBS), as there are currently no other curative treatment options available in the Netherlands. In case the patient rejects these invasive palliative treatment options, continuation of ASM therapy is the only actual option. After follow-up period of 2 years, this waitlist-control group is offered the SRT treatment (optional) if they still meet the inclusion and exclusion criteria.

### Comparison to current standard treatment

#### Comparison to anti-seizure medication (ASM)

According to the Dutch Epilepsy guidelines from the Dutch Neurology Society (NVN), the standard of care treatment for patients with focal epilepsy is ASM. In patients with drug-resistant epilepsy, 10% become seizure free following ASM adjustment, and an additional 10% have a greater than 75% improvement in seizure control after a median follow-up of 4 years [18].

#### Comparison to neuromodulation

VNS and DBS are both palliative treatment alternatives when patients are not eligible for open surgery. In a setting of shared decision-making with the patients rejected for open surgery, both VNS and DBS are offered as treatment alternatives. In general, VNS efficacy becomes optimal around the sixth month of treatment, and response rates (percentage of patients with at least 50% seizure frequency reduction) are achieved in approximately 45 to 65% of the patients [20], with 4% seizure freedom in adult patients with drug-resistant epilepsy [21]. These percentages are similar for the DBS patients. Compared to 55–70% seizure freedom rates 5–10 years after resective or disconnective surgery, neuromodulation is considered a palliative treatment.

### Use of co-intervention

Both in the study as well as in the waitlist-controlled arm, patients should continue their ASM. In accordance with clinical practice, the use of ASM will be considered for reduction and/or stopped after a seizure free period of at least 12 months. A specific co-intervention is not applicable. The risk of developing symptomatic edema or seizures following stereotactic radiosurgery (SRS) is poorly defined, and practitioners can prescribe corticosteroids and/or additional ASM. Therefore, we administer prescribe dexamethasone four days from start SRT, with a dose of 8–6–4–2 mg on consecutive days in patients with symptoms [22].

### Study procedures

Patients which are randomized for the intervention arm will receive SRT, which requires the preparation for radiation treatment. This includes imaging (MRI/CT), preparation of the immobilization devices, radiotherapy treatment planning, and the delivery of a single fraction SRT with a prescribed isotoxic dose of 24 Gy to the epileptogenic zone.

For all patients, the study procedures (Table 1) include the following.

**Table 1:** Study procedures

**Time point**	**Minimal follow-up**			**Prolonged follow-up**		
	**Pre-SRT**	**1 year**	**2 years**	**3 years**	**4 years**	**5 years**
Informed consent	X					
Epilepsy consultation - Seizure frequency - Seizure type - Seizure-free days - RAEC	X	X	X	X	X	X
Epilepsy - Nightly epilepsy seizure detection device (Nightwatch) - Epilepsy diary (Helpilepsy)	Continuous					
Quality of life questionnaires	X	X	X	X	X	X
Cost-effectiveness questionnaires	X	X	X	X	X	X
MR imaging	X		X		^a^	
Cognitive evaluation	X		X		^a^	
Visual field examination (only if SRT is in visual tract)	X		X		^a^	

^a^Waitlist-control group: patients which choose SRT will have additional 2-year follow-up including MRI and neurocognitive assessment

#### Epileptic seizure evaluation

Continuously, epilepsy diary information will be collected with the aid of a mobile phone application (Helpilepsy, Overijse, Belgium [23]). In addition, a nightly epilepsy seizure detection device (Nightwatch, Leiden, the Netherlands [24]) will be provided to the patients with the aim to detect eventual nocturnal seizures. The Nightwatch measurements are connected to the Helpilepsy application to provide the patient and the research team a total data capture of events. Patients are asked to annotate the type of experienced seizure. A monthly data extraction will be performed. In addition, on an annual basis, a follow-up consultation is scheduled with the trial assistant to evaluate the post-treatment severity of epilepsy and determine the RAEC.

#### Imaging

In order to carefully determine the EZ and all possible (a)symptomatic side effects, magnetic resonance imaging (MRI) will be performed for all patients before and 2 years after inclusion. The patients in the waitlist-control arm which are treated with SRT will also receive a post-treatment MRI, 4 years after inclusion (= 2 years after SRT). The MRI protocol includes T1, T2, and susceptibility weighted imaging, fluid-attenuated inversion recovery (FLAIR), inversion recovery pulse sequence (cs IR), diffusion tensor imaging (DTI), and functional MR imaging.

#### Cognitive evaluation

In order to determine possible side effects on cognitive functioning, an evaluation will be performed for all patients before and 2 years after inclusion. The patients in the waitlist-control arm which are treated with SRT will be planned for an evaluation 4 years after inclusion (= 2 years after SRT). Cognitive evaluation includes the Rey Auditory Verbal Learning Test (RAVLT [25]), Trail Making Test A and B (TMT-A; TMT-B [26]), Stroop test [27], Digit Span WAIS-IV [28], Dutch Reading Test for Adults (NLV, [29]), Fluency test (GIT [30]), and Letter Digit Substitution Test (LDST [31].

#### Quality of life

To evaluate the experienced quality of life, patients are asked to fill in questionnaires at baseline and on a yearly basis in the follow-up. Included are the EQ-5D-5L [32], AQOL-8D [33], QOLIE-31 [34], Cognitive Failure Questionnaire (CFQ [35]), and the Multidimensional Fatigue index (MFI [36]).

#### Cost-effectiveness

To be able to perform a cost-effectiveness analysis, questionnaires regarding healthcare utilization and resource use (iPCQ, iMCQ, iVICQ) will have to be completed yearly, by the patient (iPCQ, iMCQ) and the informal care giver (iVICQ).

#### Visual examination

Visual field examination will only be performed if the (to be treated) EZ is in the proximity of the visual pathway.

If there is missing information (e.g., missed appointments, questionnaires or diary information), the trial assistant will contact the patient.

### Study parameters and endpoints

The main study endpoint of this study is the reduction of seizure frequency in patients with focal DRE, resulting in a higher proportion of patients who show an improvement of at least 75% (radiotherapy-adapted Engel classification I–III) at 2 years. Secondary study endpoints are to assess the quality of life (QoL) after SRT, define safety, evaluate (serious) adverse effects, ASM use, and tolerability of SRT, and investigate the cost-effectiveness (CEA) of SRT compared to standard of care, neurocognitive, and MR imaging changes after treatment.

### Data management

Study administration and management will be performed by the trial coordinators who work at the Clinical Trial Office (CTO) Maastro. These trial coordinators will have access to the source data and the subject files and will be responsible for the archiving of all items that are necessary for reviewing the data of the study and ensuring quality control. Castor EDC will be used for the randomization, study database, and electronic questionnaires. Randomization will be based on the validated variable block randomization model within Castor EDC by the trial coordinator. A code will be attributed to each patient registered in the trial consisting of a sequential inclusion number with 3 digits and/or letters. This code will identify the patient and must be included on all case report forms. The code will be attributed by CTO and linked to the patient in a list kept by Clinical Trial Office Maastro only. This list can be viewed by the local researcher, monitor, and, if necessary, the IGJ (Inspectie GezondheidsZorg en Jeugd). The duration of the storage of study and imaging data at Maastro is 15 years. Monitoring will be done in compliance with the NFU (Netherlands Federation of University Medical Centres) Guidelines and based on “risk-based monitoring.” The monitoring will be performed by qualified monitors of Clinical Trial Office (CTO) of Maastro.

Premature termination of the study can occur when (1) the judgment of the competent METC that assessed the research is irrevocably withdrawn; (2) it appears that the continuation of the research cannot serve a scientific purpose, and this is confirmed by the METC that has given a positive assessment of the research; (3) the principal investigators are no longer able to perform the duties of principal investigator, and no substitute can be found by mutual consent; and (4) the DSMB recommends this.

### Statistical analysis

Analysis for the primary endpoint will be based on the intention-to-treat principle. Comparison between the SRT and standard of care group will be performed with a two-sided *Z*-test for proportions with significance level alpha 0.05. For this purpose, the proportion of patients reaching RAEC I–III per treatment group will be calculated and compared. Furthermore, the difference in proportions between groups will be determined and presented with the corresponding 95% confidence interval.

Statistical analysis for secondary endpoints will depend on the type of data and comparison. Differences between treatment and waitlist-control group are central to the analysis and will be presented as effect size and 95% confidence intervals. For categorical data, comparisons will be made using chi-square tests. Data from continuous variables will be checked for Gaussian distribution. Comparison of continuous variables of Gaussian-distributed groups will be performed using the independent samples *t*-test; for non-parametric analyses, the Mann–Whitney test will be adopted.

#### Quality of life (QoL)

*Quality of life (QoL)* after SRT will be calculated from the corresponding questionnaires. According to the respective scoring manuals, subscale and total scores will be calculated. Descriptive statistics for these continuous variables will be presented as described above, and comparisons between treatment groups at specific timepoints will be made.

#### Safety, (serious) adverse effects, AED use, and tolerability of SRT

*Safety, (serious) adverse effects, AED use, and tolerability of SRT* are all categorical measures that are registered at predetermined timepoints and presented as proportions (i.e., prevalence of adverse effects) and 95% confidence intervals. Comparisons between both treatment groups will be done using chi-square tests and will be presented as effect size and 95% confidence interval.

#### Cost-effectiveness

Decision analytical modeling will be used to estimate the cost-effectiveness of SRT compared to standard of care for adult patients with drug-resistant localized epilepsy not eligible for surgery. A state-transition model will be developed, to estimate and compare costs and (quality adjusted) life years for both treatment options. This decision analytic model will be developed in accordance with (inter)nationally recognized good practices [37], relevant technical support documents of the National Institute for Health and Care Excellence, and the Dutch Pharmaco-economic guideline (e.g., lifetime time horizon). To inform the model, utility (EQ-5D instrument) and resource use will be measured during study follow-up, while unit costs will be based on the Dutch cost guideline, and unrelated medical costs will be incorporated using the PAID tool. If required, published literature, clinical guidelines, hospital financial administration, and/or expert opinion will be used to inform input parameters. The structure and parameter estimates will be validated using expert consultation and uncertainty will be estimated using deterministic and probabilistic sensitivity analyses.

#### Neurocognition

For neuro-cognitive test variables, the Reliable Change Index (RCI) will be calculated by dividing the difference between pre-treatment (baseline) and posttreatment (2 year) scores by the standard error of the difference. The standard error of the difference will be defined based on the reference data from literature for each test. A RCI > 1.5 will be used to quantify as significant change. Differences in RCI between treatment and waitlist-control group will be compared and presented as effect size and 95% confidence interval.

A loss of follow-up is taken into account for 5 patients in each treatment arm. If patients withdraw from the study, patients will be referred back to the treating physician. Data collected until the time of withdrawal will be used for analysis, unless the patient decided his data must be destroyed.

### Roles and responsibilities

Maastro is the study sponsor and principal investigator of this study. Maastro has a liability insurance which is in accordance with article 7 of the Dutch Medical research Involving Human Subjects Act (WMO). Maastro also has an insurance which is in accordance with the legal requirements in the Netherlands (Article 7 WMO). This insurance provides cover for damage to research subjects through injury or death caused by the study. The insurance applies to the damage that becomes apparent during the study or within 4 years after the end of the study.

Study administration, management, and monitoring will be performed by the trial coordinators and qualified monitors within the Clinical Trial Office (CTO) Maastro.

The Radiotherapy Epilepsy Expert group (REEG) is involved in the PRECISION trial, which consists of 4 neuro-radiation oncologists experts in state-of-the-art central nervous system radiotherapy including stereotactic radiotherapy. Preliminary results of the study will be shared with the REEG to share knowledge on treatment plans, indications, target volumes, and potential adverse effects and to consult (inter)national colleagues for advice. In addition, the authors consult international neurosurgeons which have extensive experience with SRT for epilepsy.

A data safety monitoring board (DSMB) is established to assure independent trial supervision and will monitor the recruitment, the reported adverse events, and the data quality at least every 6 months or after the SRT treatment of 10 patients.

## Discussion

### Known and potential risks and benefits

It is not yet known if SRT is superior to the standard treatment (ASM and neuromodulation) for patients not eligible for curative respective or disconnective epilepsy surgery. Nevertheless, the current literature is promising, on the basis we expect that 50% of the patients in the SRT group will reach RAEC I–III after the 2-year follow-up.

For the patients in the waitlist-control group, there is no additional risk, since they receive the current standard of care treatment during the 2 year waiting/follow-up time. Given the chronic nature of the epilepsy, there is also no additional risk for sudden unexpected death in epilepsy (SUDEP).

For the patients treated with SRT, the risks are based on the location of the EZ. Information is available from treatment of patients with epilepsy, brain metastases, or benign brain lesions. Treatment-related side effects in patients with epilepsy were described in the review of Eekers et al. [17]. The most common acute side effects of SRT are headache, nausea, and/or vomiting caused by reversible intracranial edema and can be treated with corticosteroids [22]. Long-term side effects include transient neurological deficits and exacerbation of seizures, magnetic resonance imaging (MRI) changes, expected and mostly asymptomatic superior quadrantanopia (for lesions treated in the temporal lobe), ischemic events, cognitive changes [38], and radiation necrosis rarely leading to symptomatic edema or cysts requiring surgical intervention [6, 17]. If the EZ is close to the pituitary, potential risks can be estimated from the literature of treatment of patients with a pituitary tumor. Cerebral infarction has been described as a long-term complication of stereotactic radiotherapy of benign skull base tumors, mainly pituitary tumors (SIR 1.48 – 4.2) [39–41]. For patients with a pituitary adenoma, it is known that hypopituitarism can occur after conventional and stereotactic radiotherapy in 50% of the patients, 10 years after treatment [42–44]. However, in patients with a non-functional pituitary adenoma, 37–85% of the patients has already hypopituitarism at the start of diagnosis [45–47]. Deficit of hormone production after conventional radiotherapy of the pituitary with 45 Gy is 45–100% for GH, 18–30% for LH/FSH, 15–22% for ACTH, and 25% for TSH [48, 49]. Location in the eloquent cortex is associated with neurological complications in patients with brain metastasis [50, 51]; therefore, when the location of the EZ is within the eloquent areas, we can limit the dose prescription to 18 Gy. The possible side effects of SRT will be registered carefully and will be weighed against the anticipated gain in quality of life.

Possible side effects from ASM include headache, dizziness, nausea and vomiting, ataxia, fatigue, cognitive side effects, and rare, idiosyncratic reactions [52]. DBS involves the surgical placement of bilateral depth electrodes in the brain connected to a pulse-generator, reducing seizure frequency while influencing the brain networks. Potential side effects of the implantation are infection, seizures, cerebral hemorrhage, and stroke. Potential effects from the stimulation are sleep disturbance, mood disturbance, headache, confusion, and difficulty concentrating [53]. VNS is performed by implantation an electrode coil around the left cervical vagus nerve connected with a subcutaneous lead to the pulse generator in a subcutaneous thoracic pocket reducing the seizure frequency up to 50% in two thirds of the patients with temporary hoarseness, bradycardia, infection, paresthesia, and dysphonia as known side effects [54].

### Explorative treatment planning

**Fig. 3:**
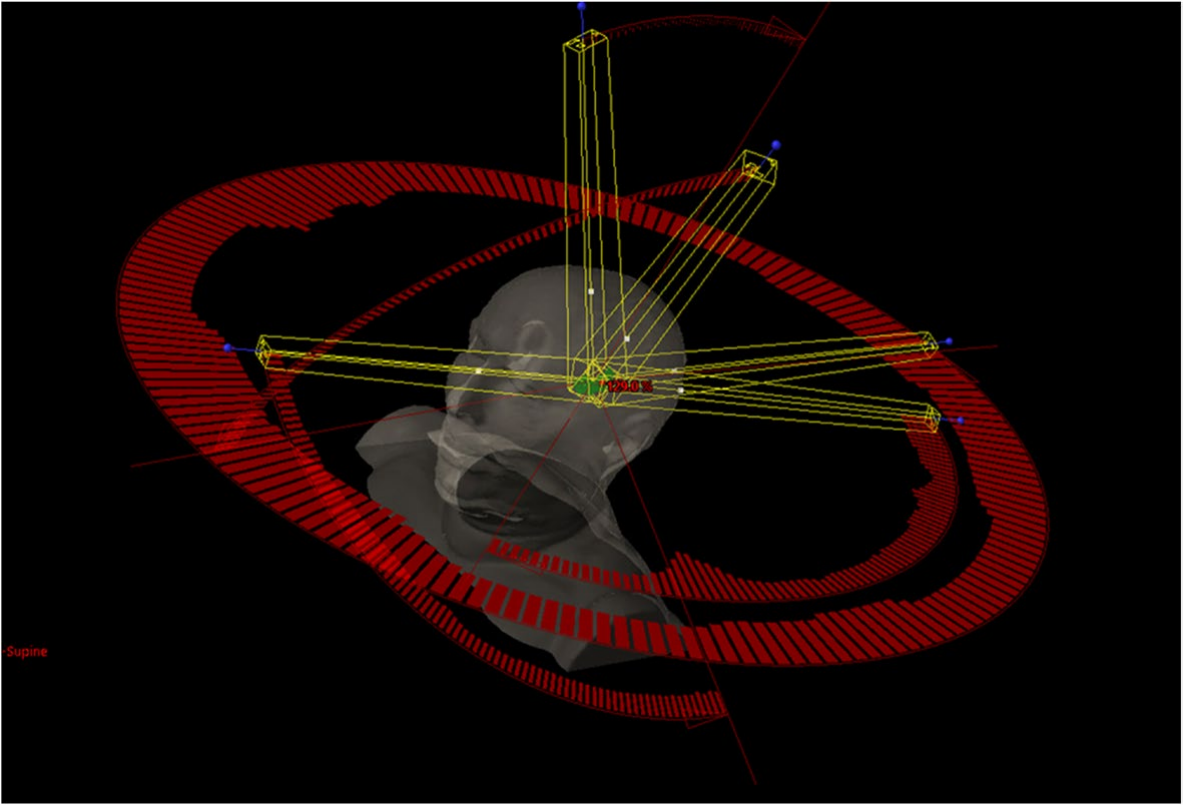
Example of a treatment plan for a simulated epileptic zone in the CT of a patient

In an anonymized patient case, 10 EZ locations were simulated, and SRT treatment plans were created to evaluate the feasibility of treatment planning. All EZ locations range from 0.14 to 5.94 cc with locations, e.g., hippocampus, amygdala, and small lesions, representing periventricular heterotopia or EZ anywhere in the brain (Fig. 3). An adequate treatment plan with a coverage of 99% of the EZ and a V_12Gy_ ranging from 0.7 to 10.7 cc was possible (Fig. 3). We created 5 templates with 4 or 5 non-coplanar arcs. The developed templates can be individually adjusted, introducing more or less beams, or change the rotation angle of the gantry or the table couch to further optimize the treatment plan.

## Conclusion

To summarize, based on the promising results of SRT in patients with epilepsy in the literature, the relative low risks of complications and the feasibility to perform this treatment in a safe manner provide the basis for the PRECISION trial. In this study, the evidence will be gathered on the relative treatment and cost-effectiveness of SRT for patients with drug-resistant, focal epilepsy, non-eligible for open surgery.

## Trial status

Recruitment for the PRECISION trial has started on 1 December 2023. The approximate date when recruitment will be completed is Q2 of 2027. The current PRECISION METC Protocol date is 4 September 2023 and has the METC MUMC + identification number: NL84071.068.23.

### Supplementary Information

Supplementary Material 1.

## Data Availability

The datasets analyzed during the current study and statistical code are available from the corresponding author on reasonable request, as is the full protocol.

## Abbreviations

PRECISION: *PRECISION* Radiotherapy to treat *Epilepsy*
SRT: Stereotactic radiotherapy
EZ: Epileptogenic zone
ASM: Anti-seizure medication
DRE: Drug-resistant epilepsy
LINAC: Linear accelerator
VNS: Vagal nerve stimulation
DBS: Deep brain stimulation
RAEC: Radiotherapy-adapted Engel classification
